# Development of Piezoelectric Inertial Rotary Motor for Free-Space Optical Communication Systems

**DOI:** 10.3390/mi15121495

**Published:** 2024-12-14

**Authors:** Laurynas Šišovas, Andrius Čeponis, Dalius Mažeika, Sergejus Borodinas

**Affiliations:** 1 Department of Aeronautical Engineering, Antanas Gustaitis’ Aviation Institute, Vilnius Gediminas Technical University, LT-10223 Vilnius, Lithuania; laurynas.sisovas@vilniustech.lt; 2 Institute of Mechanical Science, Faculty of Mechanics, Vilnius Gediminas Technical University, LT-10223 Vilnius, Lithuania; 3 Department of Information Systems, Faculty of Fundamental Sciences, Vilnius Gediminas Technical University, LT-10223 Vilnius, Lithuania; dalius.mazeika@vilniustech.lt; 4 Department of Applied Mechanics, Faculty of Civil Engineering, Vilnius Gediminas Technical University, LT-10223 Vilnius, Lithuania; sergejus.borodinas@vilniustech.lt

**Keywords:** inertial piezoelectric motor, low Earth orbit, space, angular motion

## Abstract

This paper presents the design, development, and investigation of a novel piezoelectric inertial motor whose target application is the low Earth orbit (LEO) temperature conditions. The motor utilizes the inertial stick–slip principle, driven by the first bending mode of three piezoelectric bimorph plates, and is compact and lightweight, with a total volume of 443 cm^3^ and a mass of 28.14 g. Numerical simulations and experimental investigations were conducted to assess the mechanical and electromechanical performance of the motor in a temperature range from −20 °C to 40 °C. The results show that the motor’s resonant frequency decreases from 12,810 Hz at −20 °C to 12,640 Hz at 40 °C, with a total deviation of 170 Hz. The displacement amplitude increased from 12.61 μm to 13.31 μm across the same temperature range, indicating an improved mechanical response at higher temperatures. The motor achieved a maximum angular speed up to 1200 RPM and a stall torque of 13.1 N·mm at an excitation voltage amplitude of 180 V_p-p_. The simple and scalable design, combined with its stability under varying temperature conditions, makes it well suited for small satellite applications, particularly in precision positioning tasks such as satellite orientation and free-space optical (FSO) communications.

## 1. Introduction

The advent of small satellites has revolutionized the sector of space exploration and communications, marking a significant shift toward more cost-effective, accessible, and versatile space missions [1,2,3]. These satellites are increasingly popular due to their low cost [4], shorter development cycles [5], and the ability to form constellations that can deliver comprehensive data coverage [6]. Their applications span from Earth observation and scientific research to national security and telecommunications, reflecting their growing importance in the modern space industry [7,8,9]. Despite the wide application range of small satellites, their most notable impact is to communication and data transfer areas. Therefore, in conjunction with the rise of small satellites, free-space optical (FSO) communications have emerged as a critical technology that offers high-speed data transmission without reliance on physical media [10,11]. FSO communications present numerous advantages over traditional radio frequency (RF) communications, including greater bandwidth, enhanced security, and reduced risk of interference [12,13]. However, technology also faces significant challenges, notably the need for precise pointing to maintain the alignment of communication links over vast distances [14]. This requirement underscores the importance of robust and accurate actuation systems. Currently, the majority of small satellite missions with FSO or other loads rely on brushless direct current or stepper motors, for orientation and positioning tasks. Although commonly used, these motors often fail in terms of precision, responsiveness, and power efficiency, particularly in the vacuum of space [15,16,17]. Furthermore, electromagnetic motion sources have relatively high mass and demand on gears or reduction systems and do not exhibit self-locking ability [18]. Furthermore, their susceptibility to magnetic interference can degrade performance in critical applications and create an additional motion error [19,20,21]. Therefore, it can be found that the usage of electromagnetic motion sources can be one of the limiting aspects for the further development of small satellites and their systems. These aspects create a demand to replace electromagnetic motion sources. One of the most promising motion sources that could replace electromagnetic ones are piezoelectric actuators and motors [22].

Piezoelectric actuators and motors offer numerous advantages, including high precision, electromagnetic cleanliness, low power consumption, and self-locking ability [23,24,25]. Inherent scalability and low power demand, low mass, the possibility of direct integration to systems, free operation of gear or reduction systems, as well as direct load driving make them particularly suitable for small satellites [26]. Furthermore, piezoelectric actuators and motors can be designed to drive loads with several degrees of freedom and provide linear and angular motions without changes in their structural design [27]. Therefore, recent developments in piezoelectric motion sources have led to the innovation of rotary piezoelectric motors, which provide precise rotational movements essential for accurate alignment required in FSO communications or at other tasks.

Yang et al. explored the potential of a novel ultrasonic traveling wave rotary motor, which includes a piezoelectric backup function designed to ensure operational continuity under various conditions. This motor uses the elastic properties of materials to generate rotary motion through traveling wave vibrations. The backup piezoelectric mechanism provides redundancy, crucial for applications where failure is not an option, such as in satellite orientation systems. Their study highlighted the characteristics of motor motion, including high precision in angular displacement and control of rotational speed. The traveling wave vibrations enabled for continuous and smooth motion, minimizing energy losses and maintaining stability. The motor also demonstrated high torque density and rapid response times, particularly under varying loads, with rapid acceleration to desired speeds, making it highly effective for precise positioning in space environments [28].

Chen et al. developed a rotary piezoelectric motor using a rectangular frame with four actuators, enabling direct conversion of vibrational energy into rotational motion. This design is noted for its compactness and efficiency, attributes that are particularly beneficial in space applications where equipment space is at a premium. The actuators are activated by synchronized sawtooth signals, optimizing the response time and reducing energy consumption, thus aligning with the energy efficiency requirements of modern space missions. Motor motion characteristics include precise control of rotational speed, with smooth acceleration curves and minimal vibration during operation. The design demonstrated excellent torque generation and fine-tuned angular displacement control, essential for applications requiring high-precision positioning in space. Furthermore, the motor exhibited rapid response to changes in the signal, ensuring stable performance under various operational loads and environmental conditions, further underscoring its suitability for space missions [29].

He et al. introduced a resonant inertial impact rotary piezoelectric motor featuring a self-clamping structure, which dynamically adjusts the contact force to optimize torque and enhance efficiency. The design focuses on high-speed rotational capabilities and precision, facilitated by the motor resonant operation on piezoelectric vibrations. Such features are essential for high-stakes applications in space exploration and satellite maneuvering, where precise and reliable actuation is critical [30].

Therefore, on the basis of the state of the art, the inertial piezoelectric motors stand out among piezoelectric actuation systems due to their ability to combine high precision with simple mechanical design and excitation schematics [31,32]. Compared to traveling wave motors, inertial motors exhibit lower mass, volume, and structural complexity which are critical parameters for space applications [33]. Moreover, inertial motors and actuators, despite their design, can be driven in two modes, that is, resonance and non-resonance, which expands the flexibility of their application [34]. Finally, the simplicity in achieving rotary and linear motions without complex assemblies makes inertial motors and actuators highly adaptable to applications with constraints of size and weight [35].

This article presents both numerical and experimental studies of the piezoelectric inertial rotary motor whose target application is FSO systems. The main advantages, compared to the state of the art, of proposed motor design are small size and mass, simple and well-scalable design, and the possibility of direct motor integration to the printed circuit board (PCB). These physical advantages of the proposed motor are archived via a novel approach of motor structural design which allows composing three piezoelectric bimorph plates to one structure via a connection cylinder with reduced structural stiffness. Such composition of the motor allows to increase displacement amplitudes of contacts and, as a result, the dynamic characteristics of the whole motor. Finally, the main goal of this work is to investigate how a lightweight piezoelectric inertial motor operates under temperature conditions that are close to conditions that occur during FSO system operation and how these conditions impact the motor performance and motor excitation conditions. The numerical and experimental investigations were performed and have shown that the proposed motor design is capable of proposing stable and suitable motion characteristics in the temperature ranges which occur during FSO systems operation. 

## 2. Motor Design and Operation Principle

**Figure 1:**
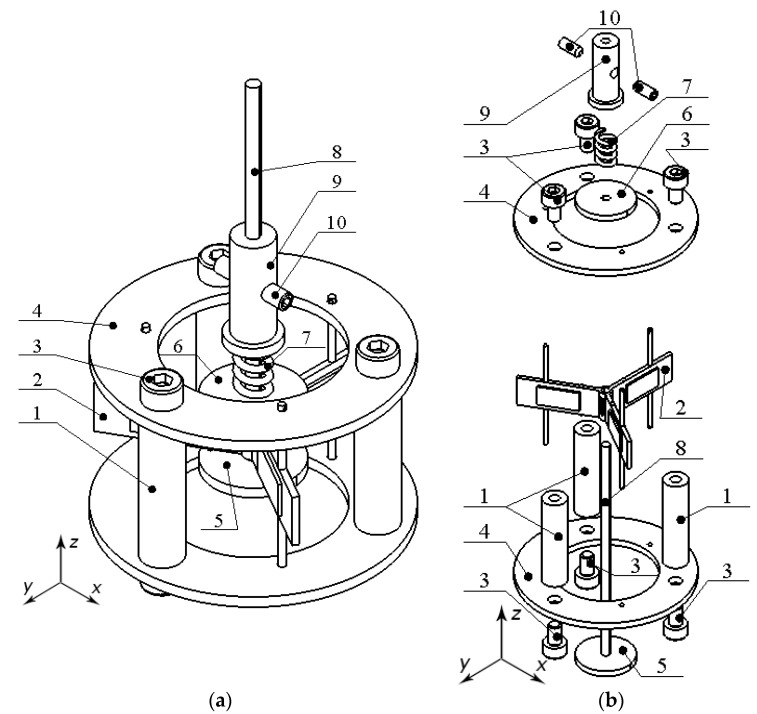
Design of the motor: (**a**)—assembled view; (**b**)—exploited view; (1)—support of printed circuit boards; (2)—stator; (3)—clamping bolts; (4)—printed circuit boards; (5)—bottom disc-shaped rotor; (6)—top disc-shaped rotor; (7)—preload spring; (8)—axis; (9)—spring clamper; (10)—spring clamper fixing bolts.

The motor is composed of a stator that is clamped, via stator supports, between two printed circuit boards that are additionally connected to each other by three supports. The top and bottom surfaces of the stator have three alumina oxide contacts, each of which is used to preload disc-shaped rotors to the stator. The preload force of the rotors to the alumina oxide contacts is created by the spring, whose stiffness is controlled by changing the longitudinal position of the spring clamper on the axis of the motor. The design of the motor is represented in Figure 1.

**Figure 2:**
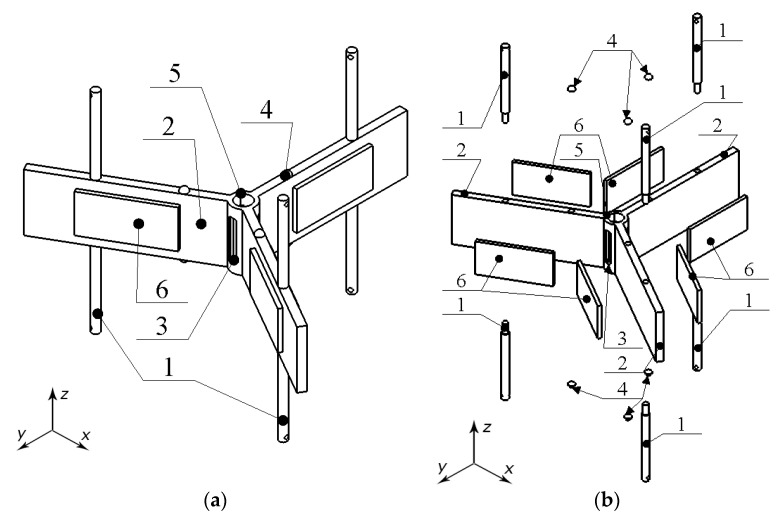
Detailed design of the stator: (**a**)—assembled view; (**b**)—exploited view; (1)—support of the stator; (2)—elastic plate; (3)—cut in the connecting cylinder; (4)—alumina oxide contacts; (5)—connecting cylinder; (6)—piezoceramic plates.

Therefore, according to Figure 1, the design of the motor can be found to be simple and compact. The total volume of the motor is 443 cm^3^, while the footprint is 13.84 cm^2^ and the mass is 28.14 g. Therefore, considering these parameters, the motor has good physical characteristics for space applications. However, to fully represent the motor, a detailed stator design is shown in Figure 2.

As can be found in Figure 2, the stator is composed of three elastic rectangular plates on both surfaces of which are placed piezoceramic plates and, by this way, piezoelectric bimorphs are composed. 

**Figure 3:**
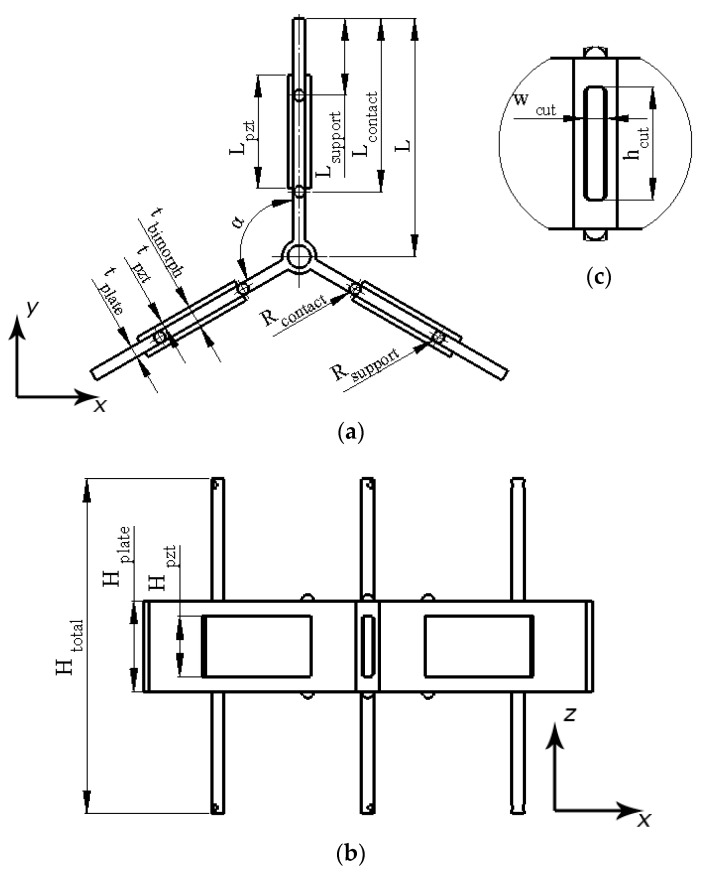
Detailed sketch of the stator: (**a**)—top view; (**b**)—side view; (**c**)—detailed view of cut at connecting cylinder.

The piezoelectric bimorph plates are composed to one structure via the connecting cylinder, i.e., one of the ends of the piezoelectric bimorph plates is connected to the cylinder while the angular distribution of the plates, around the vertical axis of the cylinder, is equal. Moreover, cuts are made at free surfaces of the connecting cylinder, which reduces the structural stiffness of the whole stator structure and enables the possibility to obtain higher displacement amplitudes. On the top and bottom surfaces of the stator, six alumina oxide contacts are used to transfer, via friction force, displacements of the stator to the rotors. The position of alumina oxide contacts is chosen in such a way that their vertical axis of symmetry coincides with the antinode of the first, out-of-plane, bending mode of the piezoelectric bimorph plates. Such a position of the contacts allows the transfer of the highest displacement amplitudes of the piezoelectric bimorph plates to the rotors. Finally, the supports, which are used to clamp the stator to the PCB, are placed on the top and bottom surfaces of the stator in a such way that their vertical axis of symmetry is in coincidence with the node of the first, out-of-plane, bending mode of the piezoelectric bimorph plates. Such a position of the supports allows us to reduce the structural stiffness of the whole structure. A detailed sketch of the stator and geometrical characteristics of it are given in Figure 3 and Table 1.

Therefore, operation of the motor is based on the inertial stick–slip principle, which is inducted by excitation of the first, out-of-plane, bending modes of the piezoelectric bimorph plates by a single sawtooth signal with a frequency equal or close to the resonance frequency of the vibration mode. At the first stick stage, the excitation signal is rising, and all piezoelectric bimorph plates bend sequentially to one direction.

**Figure 4:**
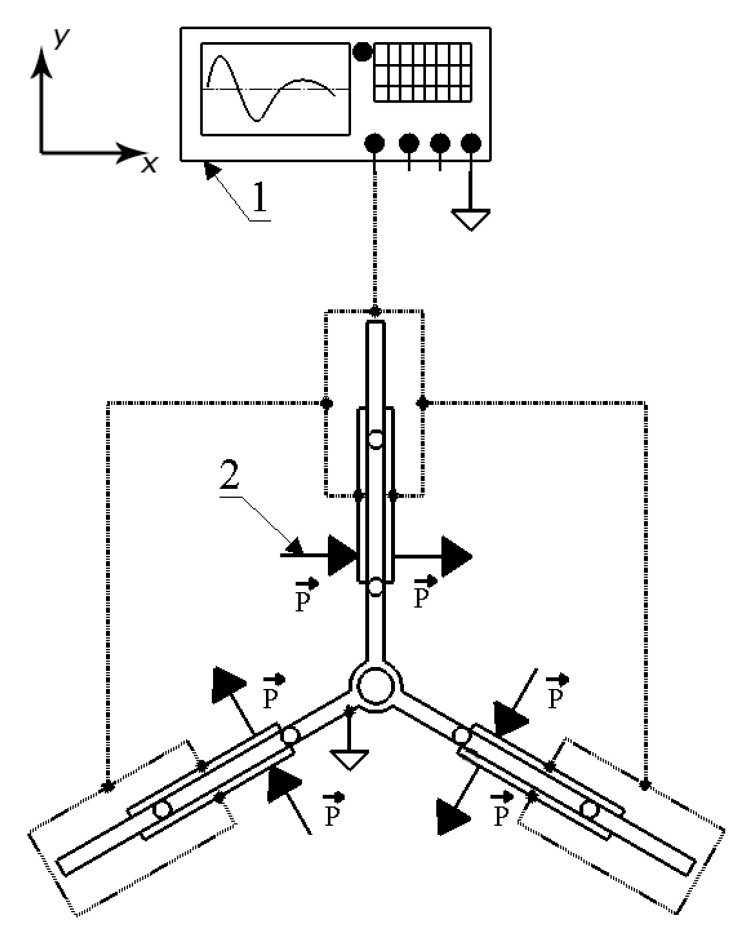
Excitation schematic of the motor: (1)—signal generator; (2)—polarization direction of piezoceramic plates.

Due to friction force, which is inducted by the preload and is higher than the force created by the plates, the motion of the plates, via alumina oxide contacts, is transferred to disc-shaped rotors, and the angular motion of the rotors is created. At this stage, the maximum angular displacement of the rotors is directly related to the maximum displacement amplitude of the piezoelectric bimorph plates. However, then the excitation signal changes its state to the opposite, during a short time range, and the slip stage occurs. During the slip stage, the motion of alumina oxide contacts has a high acceleration, whose vector is opposite to the rotor motion direction, and as a result the force created by the piezoelectric bimorph plates is higher than the friction force between the alumina oxide contacts and the rotors; as a result, the motion of the plates is not transferred to the rotors and an angular motion is not created. The continuous repetition of these stages creates the angular motion of the disc-shaped rotors. The motor excitation schematic is given in Figure 4. 

As can be found in Figure 4, the polarization directions of the piezoceramic plates are aligned to the thickness of the plates, and in each piezoelectric bimorph plate, the polarization directions of the piezoceramic plates are in series. Such arrangement of polarizations allows one to obtain single signal-based excitation of the first, out-of-plane, bending mode of the piezoelectric bimorph plates and, as a result, motion generation. Moreover, to control the direction of angular motion, the excitation signal phase must be changed by π at the level of signal generation, which allows it to be controlled electronically.

## 3. Numerical Investigation of Motor

Numerical investigations were performed to confirm the operation principle and indicate the mechanical, electromechanical, and thermal characteristics of the motor. In addition, an optimization study was performed to obtain optimal geometric and mechanical characteristics of the motor. The Finite Element Model (FEM) was built using COMSOL Multiphysics 5.6 software with respect to the geometrical characteristics, which are represented in Figure 3 and Table 2. The boundary conditions were established as follows: the stator supports, at the free ends, were rigidly fixed. Electrical boundary conditions as well as polarization directions of the piezoceramics were established with respect to the schematic shown in Figure 4. Finally, the material properties were included in the model. The stator was set as made of C17200 beryllium bronze while the piezoelectric plates were set as made of hard NCE81 piezoelectric material, and finally, the contact elements were set as made of alumina oxide material. The characteristics of the materials used to build the model are given in Table 2.

The first step of the numerical investigation was dedicated to finding the optimal geometric parameters of the stator. For this purpose, a frequency domain study was performed in the frequency range of 7 kHz to 20 kHz with a step of 10 Hz, while the excitation signal was set at 100 V_p-p_. The optimization of the motor stator was carried out with the goal of maximizing the displacement amplitudes of the contact elements in the *u_y_* direction of each bimorph plate by finding the correct combination length (*L*) and height (*h*) of the elastic plate (Figure 2—(2)). The displacement amplitude was used as an optimization criterion. The definition of the optimization problem is given in Equation (1).

(1) $$\begin{array}{c} max\\ L,h \end{array}u_{y}\left(L,h\right);$$

subject to

(2) $$L_{min}\leq L\leq L_{max};$$

(3) $$h_{min}\leq h\leq h_{max}.$$

here, *u_y_* is the displacement amplitude in the *y*-direction; *h* is the height of the elastic plate; *L* is the length of the elastic plate; *L_min_* and *L_max_* are the lower and upper limits for the plate length; *h_min_* and *h_max_* are the lower and upper limits for the height of the plate.

**Figure 5:**
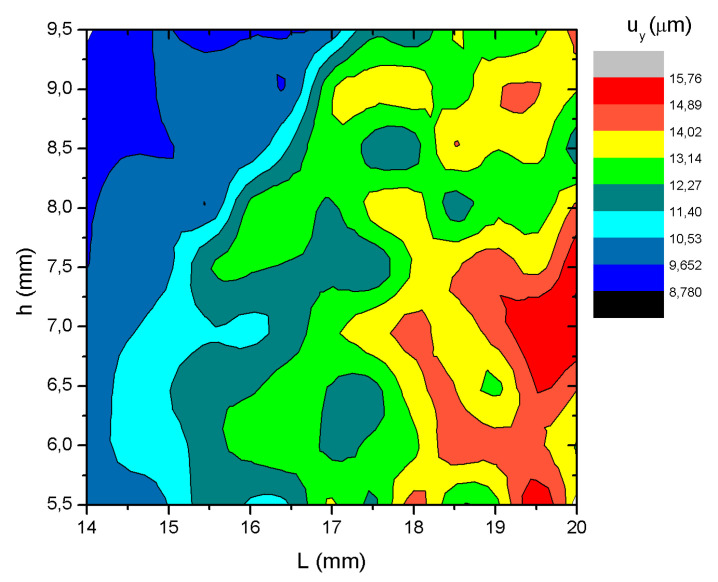
Displacement amplitudes of the motor while different geometrical parameters are used.

Therefore, in order to perform the optimization study, *h* and *L* were varied within specific ranges. Specifically, *h_min_* and *h_max_* were set to 5.5 mm and 9.5 mm, with an increment step of 0.5 mm. Similarly, *L_min_* and *L_max_* were set to 14 mm and 20 mm, with an increment step of 0.5 mm. The results of this study are depicted in Figure 5.

Upon analysis of the results shown in Figure 5, it was found that the maximum displacement amplitude in the y-direction was achieved when h was equal to 7.5 mm and *L* was equal to 20 mm, reaching a peak of 15.76 μm. On the other hand, there were other combinations of parameters that propose similar displacement amplitudes, such as *h* equal to 7 mm and *L* equal to 19.5 mm with displacement amplitude of 14.61 μm and *h* equal to 6.5 mm with *L* equal to 19 mm displacement amplitude reaching 13.16 μm. So, these results suggest that the optimal geometric parameters to maximize use of the motor have been identified and can be used for further investigations. 

**Figure 6:**
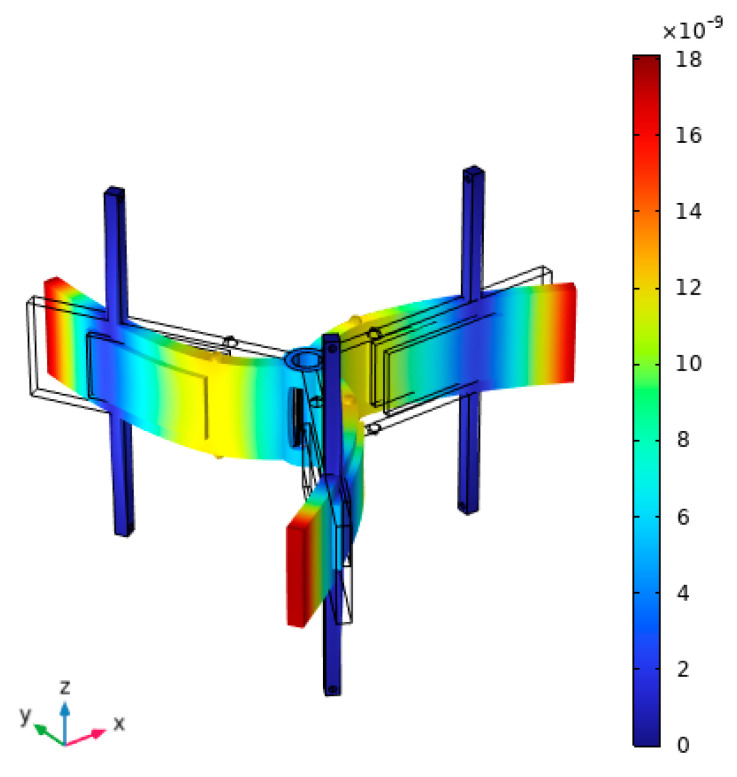
The modal shape of the stator at 12.82 kHz.

The next phase of the numerical investigation focused on conducting a modal analysis of the motor. For this study, the geometric parameters and material properties established during the optimization process were applied to the numerical model, while other physical and geometric parameters were established as given in Table 1 and Table 2. The primary objective was to determine the modal shape and its natural frequency suitable for the generation of angular motion using the inertial stick–slip principle. The results of the calculations are given in Figure 6.

The results of the modal analysis, as shown in Figure 6, revealed that the modal shape of the stator suitable for motor operation via the inertial operation principle is 12.82 kHz. The bimorph plates at this natural frequency vibrate at the first bending mode which allows to obtain the inertial stick–slip principle while a sawtooth signal is applied to the plates. Moreover, Figure 6 shows that the stator supports are placed at the nodes of the vibration mode nodes. Such positioning of the supports allows to reduce structural damping of the whole stator. Moreover, as the figure shows, alumina oxide contacts are at the antinodes of the vibration mode. This allows the proper transfer of the vibration amplitudes of the stator to the rotor and, as a result, the proper performance of the whole motor. Overall, considering the results of the modal analysis, it can be stated that the operation of the motor in the first bending mode, as the well as the positions of the supports and contacts, will ensure proper operation of the motor.

**Figure 7:**
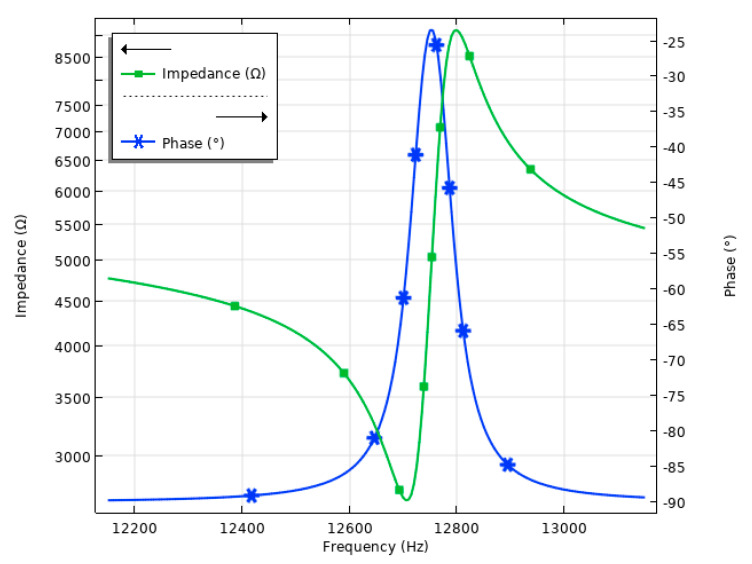
Impedance– and phase–frequency characteristics of the stator at 20 °C.

The next stage of the numerical investigation was dedicated to the calculation of the impedance and phase frequency characteristics of the motor at a temperature of 20 °C. For this purpose, a frequency domain study was conducted over a range from 12,100 to 13,200 Hz, with a step size of 5 Hz, while other boundary conditions were set as in the previous case. The results of the calculations are given in Figure 7.

As can be observed in Figure 7, the resonance frequency of the motor was identified at 12,660 Hz while the anti-resonance frequency is at 12,800 Hz. A slight mismatch of 45 Hz or around 0.5% between the resonance and natural frequencies can be attributed to discrete frequency steps used in the frequency domain analysis. Furthermore, the effective electromechanical coupling coefficient *k_eff_* of the motor was calculated and found to be 0.1084 (Equation (4)).

(4) $$\frac{k_{eff}^{2}}{1-k_{eff}^{2}}=\frac{f_{a}^{2}-f_{r}^{2}}{f_{r}^{2}}$$

Here, *k_eff_* is the electromechanical effective coupling; *f_a_* is the antiresonance frequency; and *f_r_* is the resonance frequency.

**Figure 8:**
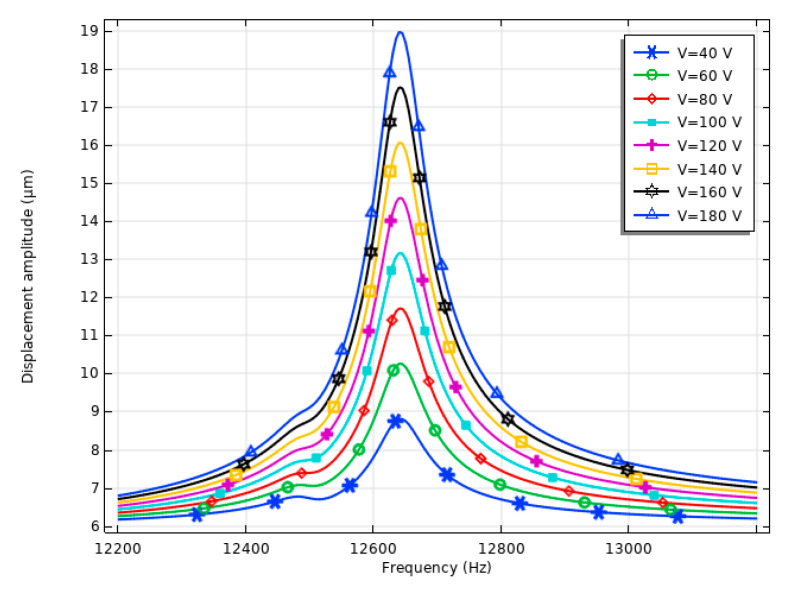
Displacement–frequency characteristics of the stator.

The next stage of the numerical study aimed to investigate the displacement–frequency characteristics while various excitation signal amplitudes were applied to the stator. The analysis was carried out over a frequency range of 12,200 Hz to 13,200 Hz, with a step size of 5 Hz, while varying the excitation signal amplitude from 40 V_p-p_ to 180 V_p-p_ in increment steps of 20 V_p-p_. The results of the calculations are given in Figure 8.

**Figure 9:**
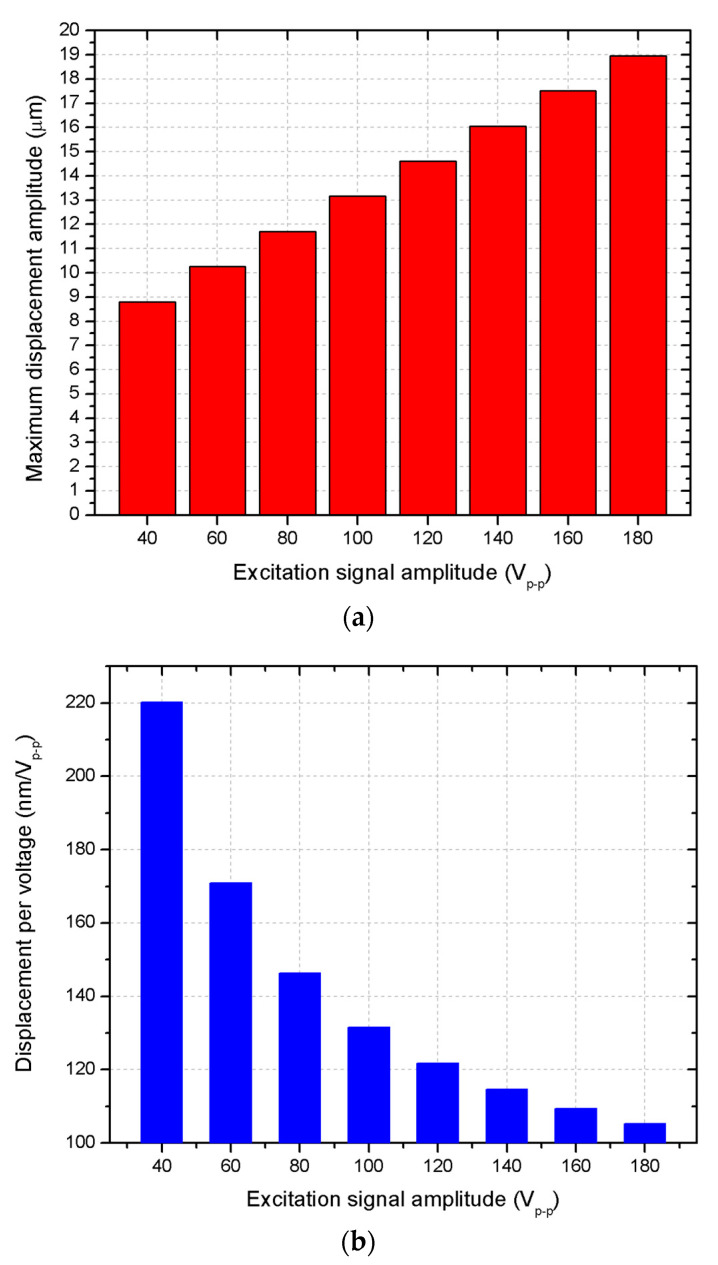
Summary of motor displacement–frequency characteristics: (**a**)—maximum displacement values; (**b**)—maximum displacement values ratio to excitation signal amplitude.

The simulation results, shown in Figure 8, demonstrate that the displacement amplitudes of the motor are directly related to the amplitude of the excitation signal. At the lowest excitation voltage of 40 V_p-p_, the displacement amplitude reached approximately 8.81 μm or 220.21 nm/V_p-p_. On the contrary, at the maximum excitation amplitude of 180 V_p-p_, the displacement amplitude peaked at 18.96 μm or 105.36 nm/V_p-p_. These results indicate that the motor displacement response scales predictably with the applied voltage, ensuring stable and controllable motion across a wide range of excitation signal amplitudes. To fully represent the displacement characteristics of the stator, a summary of maximum displacement amplitudes as well as their ratio to the excitation signal was made. The summary is given in Figure 9.

As can be found in Figure 9, the motors exhibit stable and well-predictable displacement characteristics over the whole range of excitation signal amplitudes. This discloses that motor control and driving can be implemented via simple and cost-effective excitation and driving systems, as well as confirms that the motor is capable of delivering consistent and reliable displacement outputs, which are critical for applications requiring precise and adjustable actuation. The steady increase in displacement amplitude with higher excitation voltages suggests that the motor can be effectively tuned to achieve the desired performance by adjusting the driving voltage, providing flexibility in practical space applications.

**Figure 10:**
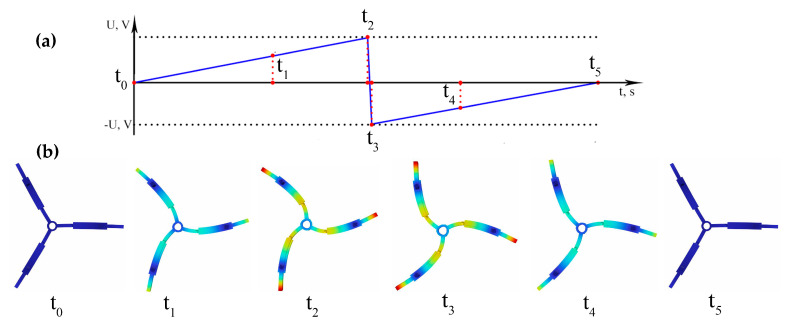
Sequence of motor vibrations during one period: (**a**)—waveform of excitation signal; (**b**)—view of motor vibrations on defined time point.

The next step of the numerical investigation was dedicated to the indication of the motor operation sequence during one period of vibration. For this purpose, a time domain study was set up with the same boundary conditions as described above while the excitation signal amplitude was set to 180 V_p-p_ and the calculation time was set to a period of resonance frequency which was equal to 78.98 µs. The results of the calculations are given in Figure 10.

Therefore, Figure 10a represents the waveform of the electrical signal used during the time domain study. The graph includes six time points, marked from *t_0_* to *t_5_,* indicating the time points when the shape of the view of the motor vibration was captured. So, during the analysis of Figure 10b, which represents the motor vibration sequence, it can be found that the vibration mode and excitation schematics are suitable for the generation of rotor angular motion through the inertial stick–slip principle i.e., the sequence shows that the motor operates in stick mode from *t*_0_ to *t*_2_, then from *t*_2_ to *t*_3_ slip mode is initiated, and from *t_3_* to *t_5_*, again, stick mode is obtained. Continuous repetition of these stages will ensure angular motion generation of the rotor. Therefore, on the basis of these results, it can be stated that motor operation at the desired configuration and principle were numerically confirmed.

**Figure 11:**
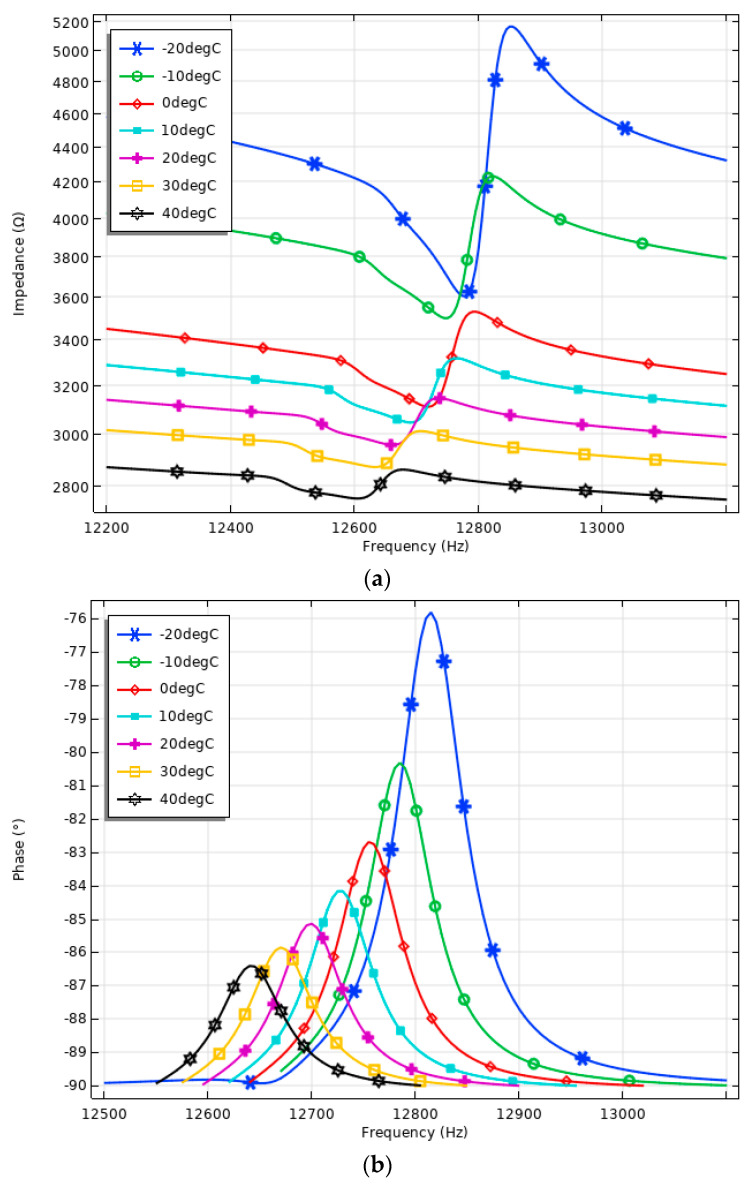
Impedance– and phase–frequency characteristics of the motor under different temperature conditions: (**a**)—impedance–frequency characteristics of the motor; (**b**)—phase–frequency characteristics of the motor.

The final step of the numerical investigation involved assessing the operation of the piezoelectric inertial motor under various temperature conditions, simulating the environment of low Earth orbit (LEO). Firstly, the numerical investigation was conducted to analyze the impedance– and phase–frequency characteristics of the motor under these temperature conditions. The study covered a temperature range from −20 °C to 40 °C, with a temperature increment step of 10 °C. For each temperature setting, a frequency domain study was performed in the range of 12,200 Hz to 13,200 Hz with a step size of 5 Hz. The results of the calculations are provided in Figure 11.

The results of the numerical analysis reveal that the impedance– and phase–frequency characteristics of the motor exhibit a clear temperature-dependent behavior. As observed in the impedance characteristics (Figure 11a), the resonant frequency and the impedance value shifts with changes in temperature. At −20 °C and −10 °C, the resonant frequencies are observed to be higher, approximately 12,845 Hz and 12,805 Hz, respectively. This increase in resonant frequency can be attributed to the reduced thermal expansion of the motor materials at lower temperatures, leading to stiffer mechanical properties and, as a result, higher structural damping. As the temperature increases to 30 °C and 40 °C, the resonant frequency decreases to around 12,685 Hz and 12,640 Hz, respectively. This decrease is due to the thermal expansion of the materials at higher temperatures, which causes a reduction in stiffness, resulting in a lower resonance frequency. The impedance peaks also become less pronounced, and the phase transitions are less sharp, indicating a decrease in the electromechanical response of the motor. At temperatures around 0 °C to 20 °C, the resonant frequency and the corresponding impedance and phase characteristics present intermediate values, reflecting a balance between the effects of thermal contraction and expansion on the mechanical properties of the materials. As depicted in Figure 11a, the impedance characteristics of the motor show significant variations across the temperature range. At lower temperatures (−20 °C and −10 °C), the impedance values near the resonance frequency are higher, indicating an increase in the electrical and structural resistance of the motor. On the contrary, at higher temperatures (30 °C and 40 °C), the impedance values drop, suggesting a decrease in electrical and structural resistance.

The phase–frequency characteristics, shown in Figure 11b, further illustrate the effect of temperature on the electromechanical characteristics. The phase shift around the resonance frequency of 12,700 Hz is more pronounced at lower temperatures, with a steep phase transition observed at −20 °C. As the temperature increases, the phase transition becomes less sharp, reflecting a change in the dynamic response of the motor. These results underscore the critical influence of temperature on motor performance, especially in environments such as LEO, where temperatures fluctuate widely. Finally, a summary of the calculated values is given in Table 3.

The resonant frequency decreases with increasing temperature, starting at 12,775 Hz at −20 °C and dropping to 12,605 Hz at 40 °C. Similarly, the impedance at resonance decreases from 3602.97 Ω at 20 °C to 2751.58 Ω at 40 °C. The phase angle also shows a significant shift, from −75.81 ° at −20 °C to −86.41° at 40 °C. The effective coupling coefficient *k_eff_* varies slightly with temperature, ranging from 0.1078 at 20 °C to 0.1072 at 40 °C, suggesting a minimal impact of temperature on the energy conversion efficiency in the motor. These results demonstrate that higher temperatures lead to lower resonant frequencies, impedance, and a more negative phase angle, which affect the motor’s performance in temperature-variable environments.

**Figure 12:**
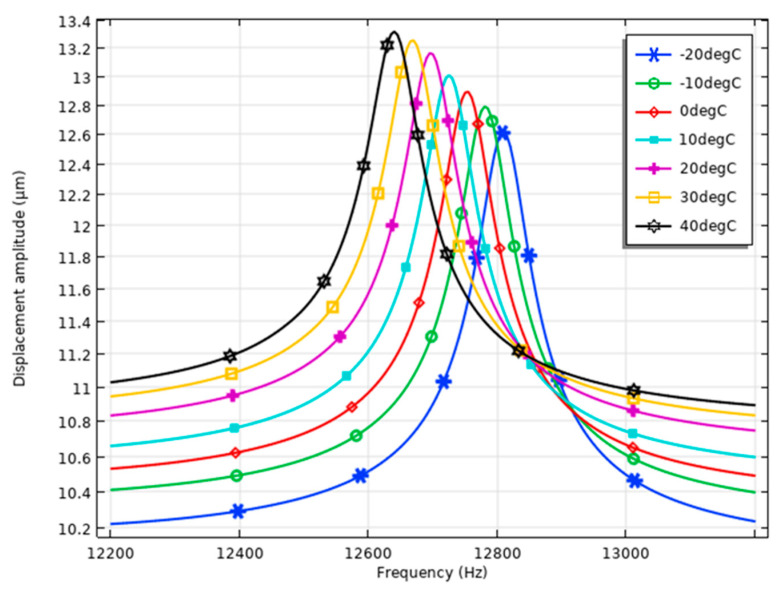
The frequency–displacement characteristics of the motor at different temperatures with excitation signal amplitude of 100 V_p-p_.

Calculations of the frequency–displacement characteristics were performed in order to indicate displacements in relation to temperatures. For this purpose, the frequency domain study was set up with the same boundary conditions as in the case before, while the excitation signal amplitude was set to 100 V_p-p_. The results of the calculations are shown in Figure 12.

The simulation results illustrate the effect of temperature variations on the displacement amplitudes and resonance frequency of the piezoelectric inertial motor. At −20 °C, the resonance frequency is approximately 12,810 Hz with a displacement amplitude of 12.62 μm (126.14 nm/V_p-p_). At 10 °C, the resonance frequency is about 12,725 Hz with a displacement amplitude of 13.01 μm (130.05 nm/V_p-p_).

At higher temperatures, the resonance frequency and displacement amplitude shift as follows: At 30 °C, the resonance frequency is approximately 12,670 Hz with a displacement amplitude of 13.25 μm (132.51 nm/V_p-p_), and at 40 °C, the resonance frequency is about 12,640 Hz with a displacement amplitude of 13.31 μm (133.14 nm/V_p-p_). As temperature increases from −20 °C to 40 °C, the resonance frequency decreases from 12,810 Hz to 12,605 Hz, a total deviation of 170 Hz, indicating temperature-dependent reductions in stiffness affecting resonance behavior.

**Figure 13:**
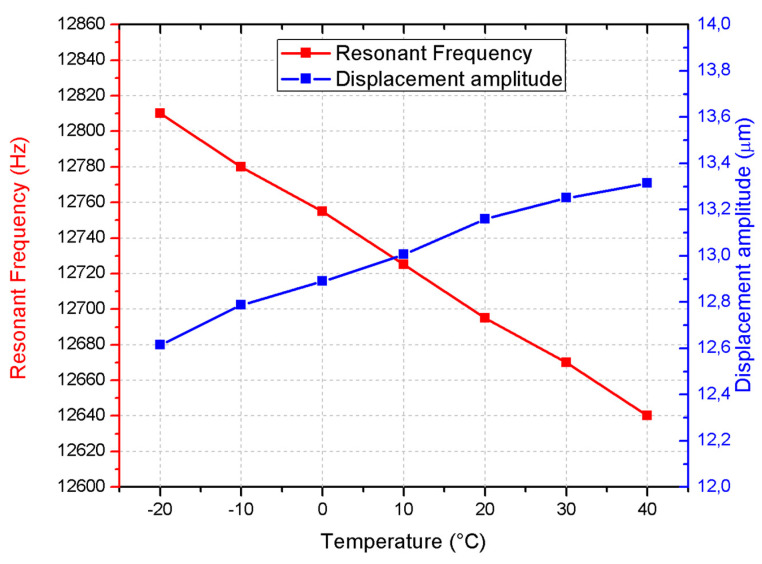
The frequency and displacement characteristics of the motor at different temperatures with excitation signal amplitude of 100 V_p-p_.

Meanwhile, the displacement amplitude increases from 12.62 μm at −20 °C to 13.31 μm at 40 °C, with a deviation of 0.70 μm, suggesting improved mechanical response at higher temperatures. These trends highlight the motor’s sensitivity to temperature, with resonance frequency decreasing and displacement amplitude increasing across the observed range, as summarized in Figure 13.

## 4. Experimental Investigation of Motor

**Figure 14:**
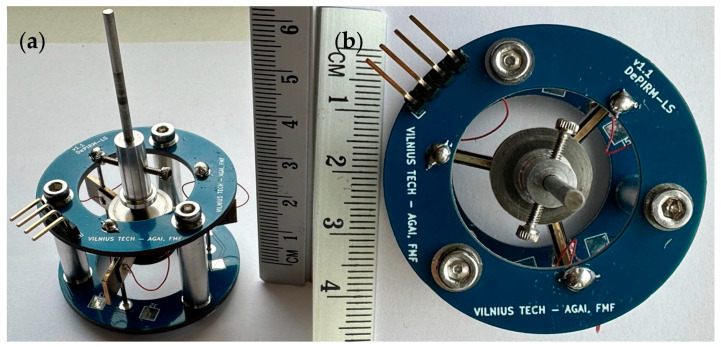
Prototype of the actuator: (**a**) side view; (**b**) top view.

Experimental investigations were conducted to verify the operation principle as the well as mechanical, electromechanical, and thermal characteristics of the motor. A motor prototype was made with strict respect to the specified geometric parameters and material properties outlined in Figure 2 and Figure 3 as well as Table 1 and Table 2. The view of the prototype is shown in Figure 14.

**Figure 15:**
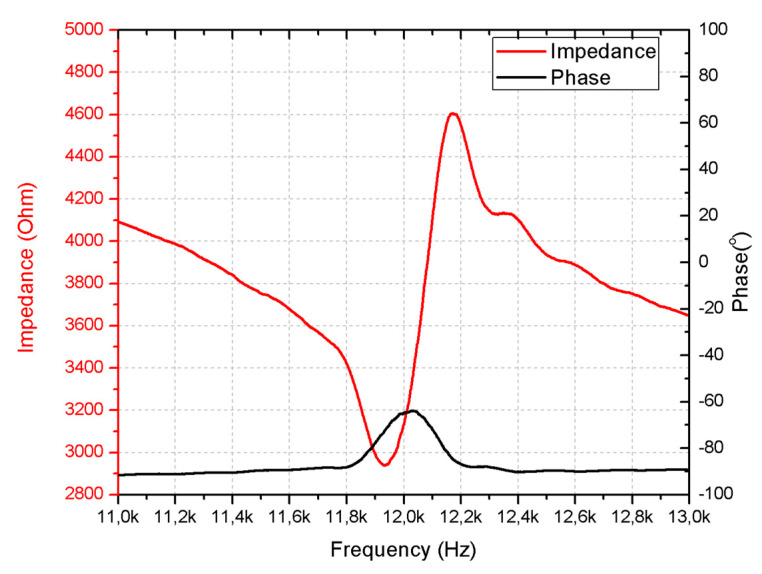
Impedance– and phase–frequency characteristics of the motor at room temperature.

First, the impedance and phase characteristics of the motor at room temperature were measured by a SinePhase 16777k impedance analyzer (SinePhase, Mödling, Austria). The results of the measurements are given in Figure 15.

As can be found in Figure 15, the resonance frequency of the motor is at 11.93 kHz while the calculated value, under the same conditions, is 12.66 kHz, which results in a difference of 6.12%, while measured and calculated impedance values are 2939 Ω and 2954 Ω; the difference in the is 0.51%. Finally, the calculations of the effective coupling coefficient have shown that the prototype has a *k_eff_* of 0.163. Therefore, the results of the experimental investigations show that the numerical model and prototype are in acceptable agreement and further investigations can be performed. 

**Figure 16:**
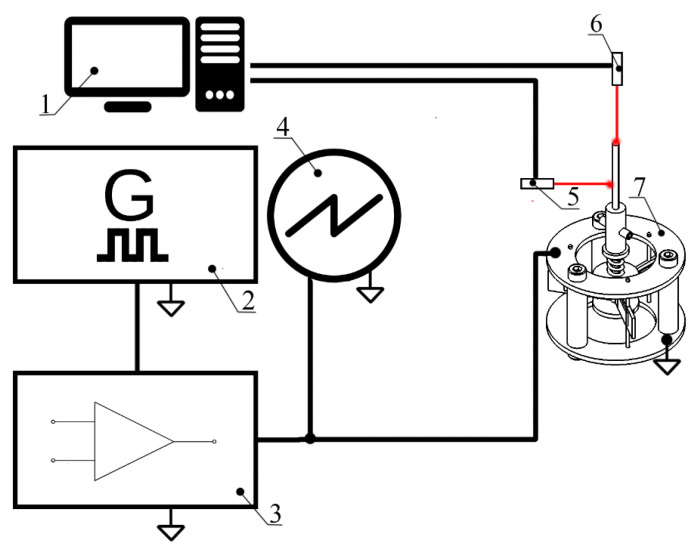
Schematic of experimental setup: (1)—a computer with data acquisition and switch control software; (2)—signal generator; (3)—power amplifier; (4)—oscilloscope; (5)—non-contact displacement sensor; (6)—non-contact tachometer; (7)—a prototype of the motor.

In order to perform experimental investigations of the motor, an experimental setup was assembled. The experimental setup is shown in Figure 16.

Therefore, the experimental setup was based on a computer with data acquisition software, a function generator WW5064 (Tabor Electronics, Nesher, Israel), a power amplifier PX-200 (Piezo Drive, Shortland, Australia), an oscilloscope DL2000 (Yokogawa, Tokyo, Japan), a non-contact tachometer CA 1727 (Chauvin Arnoux, Paris, France), and a non-contact displacement sensor optoNCDT 1420 (Micro-Epsilon, Ortenburg, Germany). 

**Figure 17:**
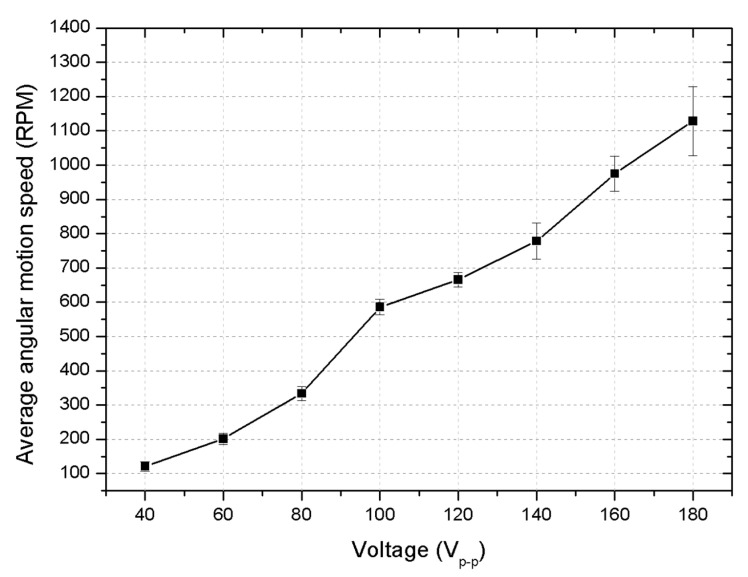
Angular motion characteristics of motor under different excitation signal amplitudes at room temperature.

Therefore, on the basis of the experimental setup, the first experimental investigation was dedicated to measurements of angular motion speed characteristics, while different excitation signal amplitudes were used to drive the motor at its resonance frequency. The results of the measurements are given in Figure 17.

As shown in Figure 17, the highest angular speed of 1178 RPM was achieved at a voltage of 180 V_p-p_. This corresponds to an angular-speed-per-voltage ratio of 6.54 RPM/V_p-p_. Conversely, under the same conditions, but at an excitation voltage of 40 V_p-p_, the angular speed reached 158 RPM, or 3.95 RPM/ V_p-p_. When analyzing the deviations, it can be observed that at low voltage levels (e.g., 40 V_p-p_), the deviations are smaller due to lower thermal effects, which results in more-stable motor performance. As the voltage increases (e.g., 180 V_p-p_), the heat generated in the motor increases significantly, causing larger deviations. This increase in thermal load can affect the consistency of the motor’s operation, leading to fluctuations in angular speed and greater measurement variability at higher voltage levels. Thus, these results indicate that the motor exhibits a stable and predictable angular speed across a wide range of voltage values, demonstrating its ability to maintain consistent performance under varying excitation conditions.

**Figure 18:**
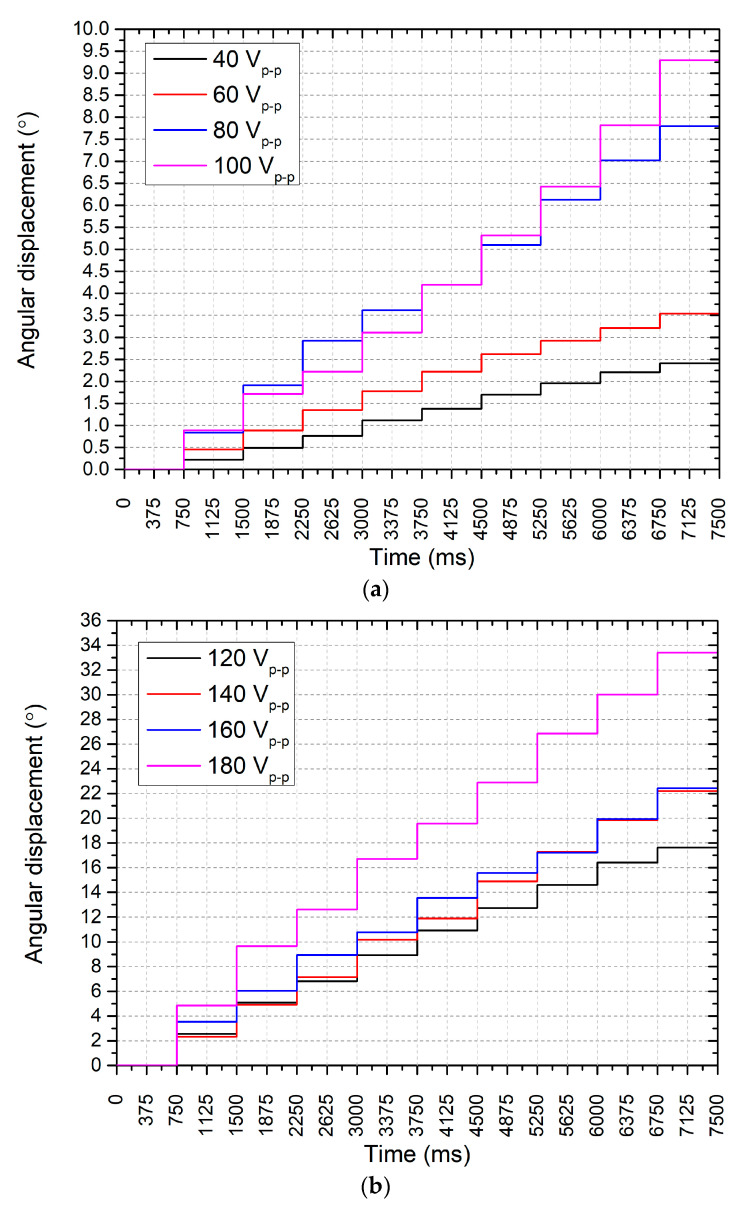
Angular motion resolution under different excitation signal amplitudes: (**a**)—in range from 40 V_p-p_ to 100 V_p-p_; (**b**)—in range from 120 V_p-p_ to 180 V_p-p_.

The subsequent investigation focused on evaluating the resolution of the angular displacement of the motor under different excitation voltages. Experimental measurements of the angular resolution of the motor were performed using a non-contact displacement sensor optoNCDT 1420 (Micro-Epsilon, Ortenburg, Germany). The burst-type sawtooth excitation signal consisting of 15 cycles at the resonance frequency of the motor during a 750 ms one-step time period was used. These excitation parameters were indicated experimentally, i.e., it was indicated that 15 cycles are the minimal number of cycles necessary to obtain angular motion at 40 V_p-p_ excitation signal amplitude, while 750 ms is the shortest time range which is necessary for proper displacement capture by the optoNCDT 1420 (Micro-Epsilon, Ortenburg, Germany) sensor. Therefore, these parameters were used to drive the motor while this arrangement of the excitation signal ensures a stable stepping-motion mode of the motor. The results are depicted in Figure 18.

In the first range (40–100 V_p-p_) in Figure 18a, the motor demonstrated a clear stepwise motion pattern, with the maximum angular displacement of 9.85° observed at 100 V_p-p_. This corresponds to a displacement–voltage amplitude of 26.9·10^−3^°/V_p-p_. At the lowest excitation voltage of 40 V_p-p_, the motor’s angular displacement was much lower, reaching 0.22°, corresponding to a displacement of 5.5·10^−3^°/V_p-p_. This result confirms that the motor’s displacement resolution is directly proportional to the excitation voltage, with higher voltages leading to greater angular displacements. The stepwise nature of the motor’s angular motion, as depicted in the graph, shows that each step corresponds to a specific angular displacement achieved by the motor at each voltage level. This precise control over angular displacement is critical for applications requiring fine positioning adjustments, such as satellite orientation or free-space optical (FSO) communication systems.

In the second range (120–180 V_p-p_) in Figure 18b, the motor performance continued to show a proportional increase in displacement with increasing excitation voltage. At 120 V_p-p_, the angular displacement reached approximately 22°, increasing incrementally with voltage, up to a maximum of around 34° at 180 V_p-p_. The linear and consistent stepping behavior across this voltage range indicates that the motor maintains stable and predictable performance under higher excitation conditions. The resolution at higher voltages also confirms that the motor can be fine-tuned for tasks that require larger angular motions while still retaining precise control. These data suggest that the piezoelectric inertial motor is capable of delivering highly controlled angular displacements across a wide range of voltages. At lower excitation voltages, the motor is suitable for applications requiring very fine, precise movements, whereas at higher voltages, it can achieve larger angular displacements for tasks that require more significant motion. The consistency of the stepwise motion at different voltage levels is particularly important for space applications where accuracy and predictability in motion control are paramount.

**Figure 19:**
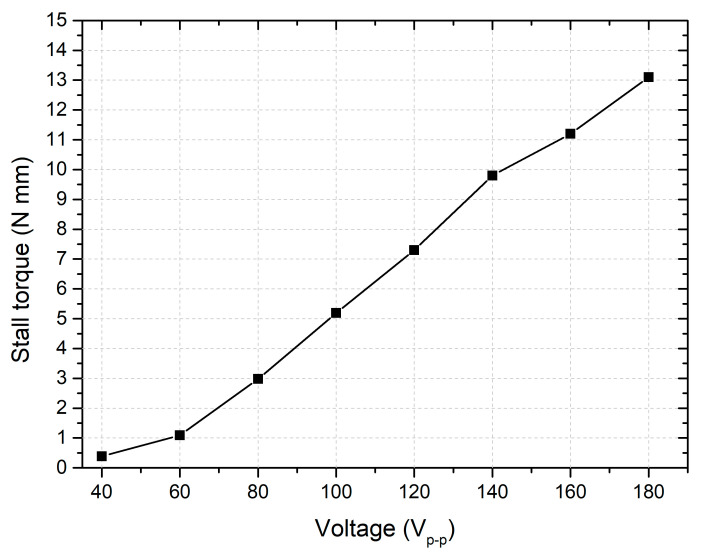
Stall torque characteristics of motor under different excitation signal amplitudes.

The next stage of the experimental investigation focused on determining the stall torque of the motor under different excitation voltages. Measurements were performed with PCE DFG N10 (PCE Instruments UK Ltd., Meschede, UK). The results of the measurements are given in Figure 19.

As shown in Figure 19, the stall torque increases proportionally with the excitation voltage. At the highest applied voltage of 180 V_p-p_, the motor achieved a maximum stall torque of 13.1 N·mm, which corresponds to a torque-per-voltage ratio of 72.7 mN·mm/V_p-p_. This value represents the maximum torque capacity, indicating its ability to handle significant loads before stalling. In contrast, at the lowest excitation voltage of 40 V_p-p_, the stall torque was measured at 0.38 N·mm, or 9.5 mN·mm/V_p-p_. This lower stall torque reflects the expected reduction in torque output with decreased voltage amplitude, demonstrating the sensitivity to changes in the driving signal. Overall, considering the stall torque–excitation signal amplitude relationship, it can be stated that the motor can provide stable characteristics that are suitable for FSO systems. 

**Figure 20:**
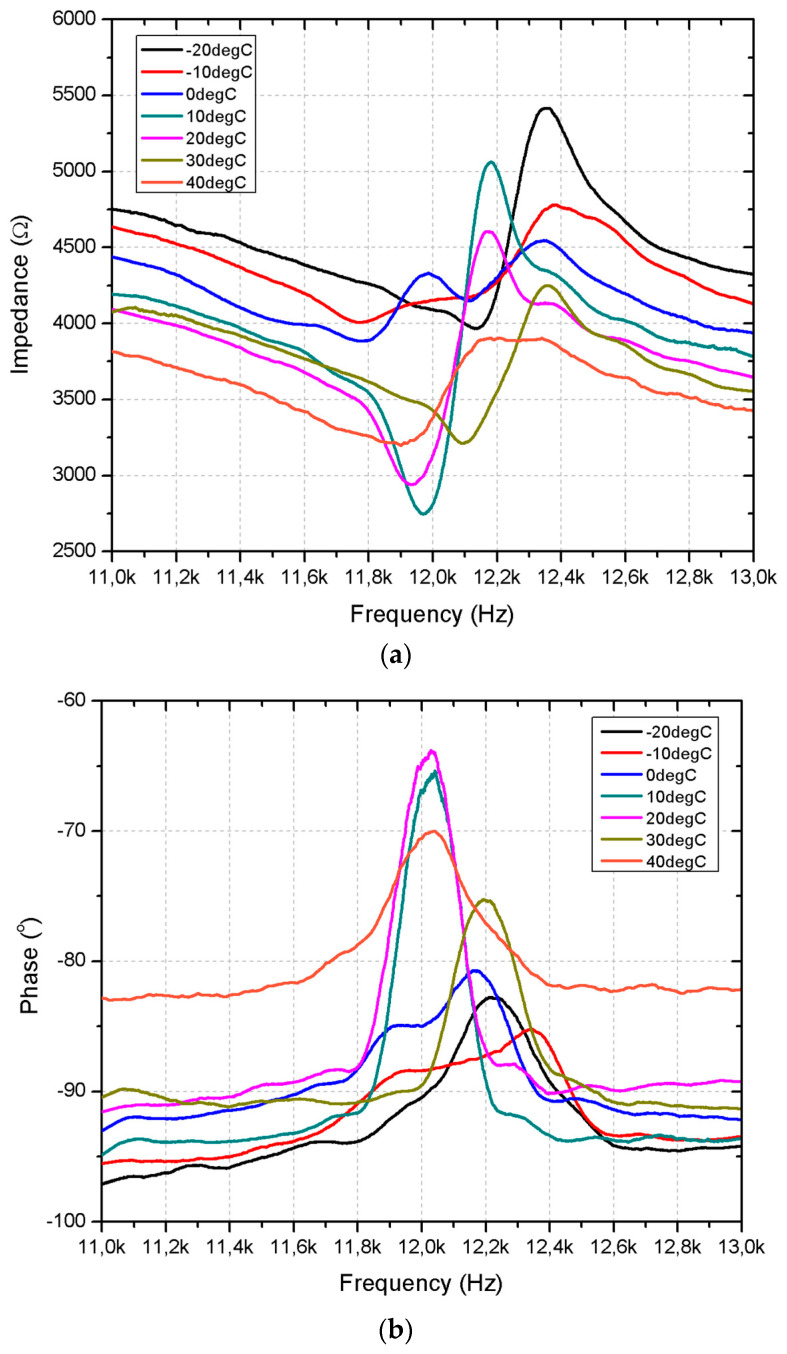
Impedance– and phase–frequency characteristics of the motor under different temperature conditions: (**a**)—impedance–frequency characteristics of the motor; (**b**)—phase–frequency characteristics of the actuator.

Finally, to simulate the thermal environment that a small satellite could encounter in low Earth orbit (LEO), the impedance and phase characteristics were measured under varying temperature conditions, from −20 °C to +40 °C. These temperatures represent the thermal fluctuations experienced by satellites in orbit as they pass through sunlight and shadow. The experiment was carried out in a RUMED XTS-T500 Ex cold–heat cabinet, and measurements, conducted using the SinePhase 16,777 k impedance analyzer (SinPhase, Mödling, Austria), are presented in Figure 20.

As shown in the Figure 20a impedance, frequency characteristics have a dependence on temperature. At lower temperatures, particularly −20 °C and −10 °C, the motor exhibits higher impedance values, with a range of 4000–4300 Ω. This behavior can be attributed to increased electrical resistance and material stiffness at colder temperatures. As the temperature increases, the impedance decreases, with the lowest values observed at 30 °C and 40 °C, where the impedance ranges from 3000–3500 Ω. 

Furthermore, the impedance behavior is strongly frequency-dependent, with noticeable resonant peaks occurring between 12 kHz and 12.3 kHz across all temperatures. Peak impedance values decrease as temperature rises, and the resonance frequency changes slightly, suggesting that the resonance characteristics are influenced by the thermal environment. This behavior is critical in space applications, where the motor’s electrical and mechanical performance must remain consistent despite rapid thermal fluctuations.

The phase–frequency response further complements the impedance findings, showing phase shifts around the resonant frequency. At −20 °C, the phase angle remains close to −85 ° for most of the frequency range, indicating that the motor operates predominantly in a reactive state with minimal energy dissipation. As the temperature increases, the phase angle becomes less negative, with a peak phase shift around −70 at 20 °C and −75° at 40 °C. This shift means that as the motor heats up, the real component of the impedance increases, suggesting that more energy is being transferred to mechanical work or dissipated as heat.

Notably, the phase deviation near resonance broadens as the temperature increases. At −20 °C, the phase shift occurs sharply around 12.2 kHz, while at 40 °C, the deviation begins at lower frequencies (around 11.9 kHz) and persists over a broader range. This behavior indicates that the motor resonance response becomes more diffuse at higher temperatures, likely due to changes in material properties affecting both the electrical and mechanical energy storage components.

**Figure 21:**
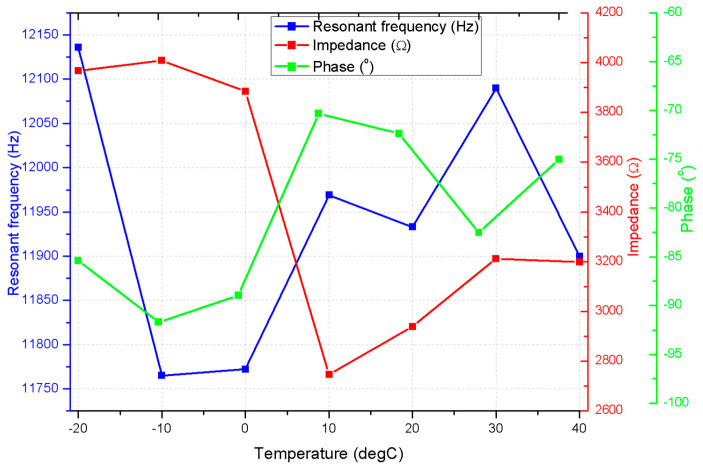
Impedance– and phase–frequency characteristics of the motor under different temperature conditions.

The combined analysis of impedance and phase characteristics (Figure 21) demonstrates that the motor’s performance is highly sensitive to temperature changes. At lower temperatures, the motor exhibits higher impedance and remains in a largely reactive state, reflecting reduced mechanical flexibility and increased energy loss due to higher electrical resistance. On the contrary, at higher temperatures, the impedance decreases and the phase shift indicates a stronger real power component, reflecting improved motor efficiency. These results have significant implications for space applications, particularly in LEO environments, where satellites experience rapid and extreme thermal cycling. The ability of the motor to maintain consistent performance in such a wide range of temperatures is critical to ensuring the reliability of satellite operations. Temperature-induced shifts in both impedance and phase characteristics highlight the need for careful thermal management strategies in the design and operation of space-based motors.

**Figure 22:**
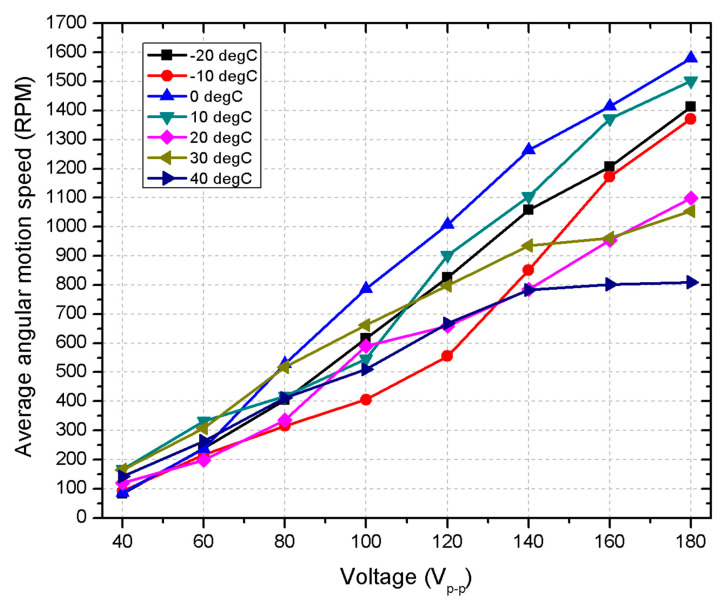
Angular motion characteristics of the motor under different temperature conditions.

To further evaluate the motor’s performance, angular motion speed measurements were conducted at varying temperatures and excitation voltages. The purpose of these measurements was to simulate the thermal and electrical conditions expected in the low Earth orbit (LEO), where small satellites experience extreme temperature fluctuations. The results, shown in Figure 22, provide information on how both temperature and voltage influence the motor’s average angular speed (RPM).

At temperatures below 0 °C, the motor shows a reduced angular speed across all voltage levels. For example, at 180 V_p-p_, the maximum angular speed reached at −20 °C is around 1400 RPM, while at −10 °C, the speed is slightly below 1400 RPM. These lower speeds can be attributed to the increase in mechanical resistance and stiffness of the motor materials at subzero temperatures, as well as to higher electrical resistance. These conditions impede the efficiency of the motor, leading to lower rotation speeds. In the temperature range from 0 °C to 20 °C, the motor achieves its highest performance. At 0 °C, the motor reaches its maximum angular speed of approximately 1600 RPM at 180 V_p-p_, the highest speed recorded in this set of experiments. The linear increase in speed with voltage in this range suggests that the motor is operating in an optimal thermal condition, where both mechanical and electrical properties are well balanced, minimizing resistance and maximizing efficiency. As the temperature increases to 30 °C and 40 °C, the motor’s angular speed begins to stall at higher voltage levels. At 40 °C, for example, the motor reaches a maximum speed of approximately 800 RPM at 140 V_p-p_, after which further increases in voltage do not result in a proportional increase in speed. This stall is likely due to thermal limitations, such as increased internal friction and material softening, which limit the motor’s ability to sustain higher speeds at elevated temperatures. This behavior highlights the motor’s sensitivity to thermal saturation, where further increases in excitation voltage do not yield improvements in angular speed.

The combined influence of voltage and temperature on the motor’s angular speed illustrates its operational efficiency under varying thermal and electrical conditions. At low temperatures, motor performance is hindered by increased stiffness of the material and electrical resistance, leading to reduced speed. On the contrary, at moderate temperatures, the motor operates at peak efficiency, achieving the highest angular speeds. However, at high temperatures, thermal effects such as material expansion and increased friction limit the speed despite the application of a higher voltage. These results are critical for space applications, particularly for LEO-operating satellites. The wide temperature fluctuations experienced in this orbit can significantly affect motor performance, as shown by the reduction in speed at both high and low temperatures.

Finally, a comparison of the key parameters of the developed inertial piezoelectric motor with the most recent state-of-the-art solutions was conducted. The results of the comparison are given in Table 4. 

Therefore, as can be found in Table 4, the proposed inertial motor exhibits a compact footprint, a lightweight design, a high angular speed, and moderate torque, making it highly suitable for applications requiring precise and efficient motion control, such as small satellite systems. While other motors excel in specific areas, such as resolution or torque, the presented motor offers a balanced performance optimized for space-oriented tasks.

## 5. Conclusions

A novel piezoelectric inertial motor was designed, developed, and tested for operation in low Earth Orbit (LEO) temperature conditions. The motor, based on the inertial stick–slip principle using the first bending mode of the piezoelectric bimorph plates, is compact and lightweight with a total volume of 443 cm^3^ and a mass of 28.14 g, which makes it highly applicable for small satellite applications. The experimental results show that the motor resonance frequency decreases from 12,810 Hz at −20 °C to 12,640 Hz at 40 °C, a deviation of 170 Hz. The stator’s displacement amplitude increases from 12.61 μm at −20 °C to 13.31 μm at 40 °C, demonstrating enhanced mechanical responsiveness at higher temperatures. The motor achieved a maximum angular speed of 1178 RPM and a stall torque of 13.1 N·mm at 180 V_p-p_. Despite these variations, the motor maintained consistent performance, confirming its mechanical stability and adaptability to temperature fluctuations in space environments. The low mass, compact design, and ability to operate reliably over a wide range of temperatures make this motor a promising solution for small satellite applications. It is particularly well suited for tasks requiring precision, such as satellite orientation and free-space optical (FSO) communications, where size, efficiency, and stability are critical.

## Figures and Tables

**Table 1:** Geometrical parameters of stator.

Parameter	Value, mm	Description
L	21	Length of elastic plate
L_contact_	15.28	Position of contact on elastic plate
L_support_	6.77	Position of stator support on elastic plate
L_pzt_	10	Length of piezoceramic plate
t_bimorph_	2	Thickness of piezoelectric bimorph plate
t_pzt_	0.5	Thickness of piezoceramic plate
t_plate_	1	Thickness of elastic plate
R_contact_	0.5	Radius of contact
R_support_	0.5	Radius of support
H_total_	27.5	Total height of stator
H_plate_	7.5	Height of elastic plate
H_pzt_	5	Height of piezoceramic plate
w_cut_	1	Width of cut at connecting cylinder
h_cut_	5	Height of cut at connecting cylinder
α	120°	Angle between piezoelectric bimorph plates

**Table 2:** Material characteristics.

Material Properties	CTS Corp NCE81	C17200 Beryllium Bronze	Alumina Oxide
Density [kg/m^3^]	7600	8360	3900
Young’s modulus [N/m^2^]	-	1.31 × 10^10^	41.9 × 10^10^
Poisson’s coefficient	-	0.34	0.2 × 10^−3^
Isotropic structural loss factor	-	0.02	-
Relative permittivity	ε_33_^T^/ε_0_ = 1020	-	-
Elastic compliance coefficient [10^−12^ m^2^/N]	S_11_^E^ = 16.00	-	-
Elastic stiffness coefficient c_33_^D^ [N/m^2^]	S_33_^E^ = 17.00	-	-
Piezoelectric constant d_33_ [10^−12^ m/V]	14.6 × 10^10^	-	-
Piezoelectric constant d_31_ [10^−12^ m/V]	225	-	-
Coefficient of thermal expansion [1/K]	−100	1.67 × 10^−5^	8 × 10^−6^
Thermal conductivity [W/(m·K)]	5 × 10^−6^	118	27

**Table 3:** Electromechanical characteristics of motor at different temperatures.

Temperature, °C	Resonant Frequency, Hz	Impedance, Ω	Phase, °	$k_{eff}$
−20	12,775	3602.97	−75.81	0.1078
−10	12,745	3498.72	−80.33	0.1080
0	12,720	3108.87	−82.69	0.1081
10	12,690	3044.27	−84.18	0.1082
20	12,660	2954.45	−85.14	0.1084
30	12,635	2868.99	−85.86	0.1048
40	12,605	2751.58	−86.41	0.1072

**Table 4:** Comparison between piezoelectric motors.

Parameter	Proposed Motor	Deng et al. [36]	Tian et al. [37]	Ma et al. [38]
Motor operation principle	Inertial	Inertial	Inertial	Traveling wave
Footprint area (mm²)	1384	2827	8850	5600
Weight (g)	28.14	50	1200	300
Angular speed (RPM)	1178 @ 180 V_p-p_	2360	3	53.86 @ 250 V_p-p_
Torque (N·mm)	13.1 @ 180 V_p-p_	1.3	93	110 @ 250 V_p-p_
Resolution (°/V_p-p_)	0.22 @ 40 V_p-p_	0.66 μrad	3.8 × 10^−5^	1.29 @ 250 V_p-p_

## Data Availability

The original contributions presented in the study are included in the article, further inquiries can be directed to the corresponding author.
